# Multicenter study of seasonal and regional airborne allergens in Chinese preschoolers with allergic rhinitis

**DOI:** 10.1038/s41598-024-54574-z

**Published:** 2024-02-27

**Authors:** Zhifeng Huang, Aoli Li, Huiqing Zhu, Junxiu Pan, Jun Xiao, Jiang Wu, Yumin Han, Lili Zhong, Xuhui Sun, Lei Wang, Liang Hu, Cuihua Wang, Xingkai Ma, Zaixia Qiao, Min Zhang, Ling Yuan, Xin Liu, Jun Tang, Yue Li, Hong Yu, Zhaobing Zheng, Baoqing Sun

**Affiliations:** 1 Department of Clinical Laboratory, National Center for Respiratory Medicine, National Clinical Research Center for Respiratory Disease, State Key Laboratory of Respiratory Disease, Guangzhou Institute of Respiratory Health, The First Affiliated Hospital of Guangzhou Medical University, Guangzhou, China; 2 Department of Child Allergy, Chenzhou First People’s Hospital, Chenzhou, China; 3 Department of Ophthalmology, People’s Hospital of Changji City, Changji, China; 4 Department of Pediatrics, Huangshi Maternity and Children’s Health Hospital, Huangshi, China; 5 Department of Allergic Reaction, Dongchangfu Maternal and Child Health Care Hospital, Liaocheng, China; 6 Pediatric Medical Center, Hunan Provincial People’s Hospital, Changsha, China; 7 Department of Pediatrics, Shengli Oilfield Central Hospital, Dongying, China; 8 Department of Pediatrics, Yunnan Diannan Central Hospital, Honghe, China; 9 Allergy Center, Changchun Children’s Hospital, Changchun, China; 10 Department of Pediatrics, Heze Municipal Hospital, Heze, China; 11 Department of Otolaryngology, Zhangjiagang First People’s Hospital, Zhangjiagang, China; 12 Pediatric Respiratory and Critical Care, Qilu Hospital of Shandong University Dezhou Hospital, Dezhou, China; 13 Dermatology, Liuzhou Municipal Liutie Central Hospital, Liuzhou, China; 14 Department of Clinical Laboratory, Chongqing Youyou Baby Women and Children’s Hospital, Chongqing, China; 15 Department of Pediatrics, Liuzhou Maternity and Child Health Hospital, Liuzhou, China; 16 Otolaryngology Department of the First People’s Hospital of Foshan, Foshan, China; 17 Respiratory Department of Dalian Women and Children’s Hospital Center, Dalian, China; 18 Department of Clinical Laboratory, Wuhan Hospital of Traditional Chinese Medicine, Wuhan, China; 19 Pediatrics Department, Huantai County People’s Hospital, Zibo, China

**Keywords:** Preschool, Allergic rhinitis, House dust mite, Pollen, Regional differences, Epidemiology, Respiratory tract diseases, Epidemiology

## Abstract

This study is nationwide multicenter epidemiological research, aimed at investigating the distribution changes and seasonal patterns of various airborne allergens among preschool children with allergic rhinitis (AR) in different regions of China, and analyzing the clinical correlation between sensitization to various airborne allergens and AR symptoms in children. Information on children was collected through standard questionnaires, and total IgE (tIgE) and specific IgE (sIgE) for 11 inhalant allergens were tested. The results showed that dust mites are the primary allergens for preschool AR children (39%). Among pollen allergens, Amb a had the highest positivity rate (8.1%), followed by Art v (7.8%). The sensitization rates for two mites peaked in May (46.9% and 40.6%). Art v peaked in August (21.5%), while Amb a had peaks in May (12.7%) and August (17.8%). The sensitization peaks for various tree pollens mainly occurred in August. In the Eastern monsoon region, the sensitization rate to mites was significantly higher than in the Northwest arid and semi-arid regions; whereas, for pollen allergens, the sensitization rates to Amb a, Pla a, Pin a, Pop d, and Bet v were significantly higher in the Northwest arid and semi-arid regions than in the Eastern monsoon region. The correlation among various tree pollens, specifically between Pla a, Pin r, Pop d, and Bet v was strong (0.63 ~ 0.79), with a cross-overlapping percentage of 53.9%. Children with multiple pollen sensitizations had higher cumulative nasal symptom scores than those negative for pollen (P < 0.01). Children with only pollen sensitization had higher cumulative rhinitis symptom scores than the all-negative group (P < 0.0001) and the mite-only sensitization group [P < 0.05], while the mite-only sensitization group also had higher scores than the all-negative group [P < 0.05], and the group sensitized to both pollen and mites had lower scores than the pollen-only group [P < 0.05]. This study indicates that sensitization to mites and grass pollens exhibits significant regional differences, with grass pollen allergies primarily occurring in autumn, sensitization to pollens in general exhibits a pronounced seasonal pattern. Moreover, pollen sensitization aggravates nasal and ocular symptoms in AR children.

## Introduction

Allergic rhinitis (AR) affects 10%-40% of the population and is a global health issue impacting both adults and children^1^. AR is defined as an inflammation of the nasal mucosa mediated by Immunoglobulin E (IgE). The main symptoms include rhinorrhea (anterior or posterior nasal drip), nasal congestion, nasal itching, and sneezing^2^.

In recent years, the incidence of AR has increased significantly. In terms of children's allergies in the world, the third phase of the International Study of Asthma and Allergies in Childhood (ISAAC) revealed that more than 80% of research centers in the 6–7 age group showed an increase in the prevalence of allergic rhinoconjunctivitis (ARC). Factors contributing to these prevalence disparities may include regional differences, age groups, lifestyle changes, genetic factors, indoor and outdoor environments, climate changes, disease awareness, and symptom management^3^. Concurrent with significant economic growth in China, changes in living standards and habits have led to a marked increase in the incidence of allergic diseases. A telephone-based study in 11 major cities found that the self-reported prevalence of AR increased from 11.1% in 2005 to 17.6% in 2011. And a questionnaire survey on allergic rhinitis in children aged 3 to 16 years from Wuhan showed that the prevalence rate of AR was as high as 25.7%^4^. The cost associated with the medical burden caused by AR and the loss and decrease in productivity caused by severe symptoms of rhinitis in patients is considerable^5,6^. Previous studies have shown that children with AR exhibit decreased academic performance, increased behavioral and attention disorders^7,8^.

Based on the timing and type of exposure as well as symptoms, allergic rhinitis (AR) can be classified into seasonal (SAR) or perennial (PAR). Overall, approximately 20% of cases are seasonal, 40% are perennial, and 40% are mixed (perennial with seasonal exacerbation)^9^. Symptoms of SAR patients usually occur during the pollination season of the main local tree grass pollen; whereas PAR patients are mainly caused by long-term exposure to dust mites, cockroaches, animal dander, and indoor mold. In China, with its vast territory and diverse geographical and natural environments, different regions have varying climatic conditions, levels of economic development, and lifestyle habits^10–12^. These factors make the prevalence, allergen types, and distribution characteristics of AR vary in different regions.

So far, there is still a lack of systematic and large-scale epidemiological investigations targeting children with AR in China. Therefore, extensive, multicenter epidemiological surveys on allergens in allergic diseases are imperative. Our study recruited preschool children diagnosed with rhinitis from multiple centers nationwide, and provide useful data for healthcare professionals to develop prevention and treatment measures. This will enable more effective and comprehensive management of AR in preschool children.

## Materials and methods

### Research object

This research is a nationwide, multi-centre epidemiological survey study. It was conducted simultaneously from January 2022 to December 2022 in 19 different study centres, including Chenzhou First People's Hospital, People's Hospital of Changji City, Huangshi Maternity and Children's Health Hospital, Dongchangfu Maternal and Child Health Care Hospital, Hunan Provincial People’s Hospital, Shengli Oilfield Central Hospital, Yunnan Diannan Central Hospital, Changchun Children's Hospital, Heze Municipal Hospital, Zhangjiagang First People’s Hospital, Qilu Hospital of Shandong University Dezhou Hospital, Liuzhou Municipal Liutie Central Hospital, Chongqing Youyou Baby Women and Children’s Hospital, Liuzhou Maternity and Child Health Hospital, The First People's Hospital of Foshan, Dalian Women and Children’s Hospital Center, Wuhan Hospital of Traditional Chinese Medicine, Huantai County People's Hospital for children attending pediatric, respiratory and allergy outpatient clinics, covering seven regions (East China, North China, Central China, South China, Southwest China, and Northwest China) and two seasons (spring and autumn) in China.

The inclusion criteria for this study were as follows: (1) preschool children aged 2–7 years; (2) Diagnosis of rhinitis based on ARIA guidelines^13^, and reported symptoms of rhinitis (itchy nose, sneezing, runny nose, or nasal congestion) lasting for more than 2 weeks, with or without conjunctivitis (eye itching, conjunctival congestion, eye swelling, and tearing); (3) were able to complete the sample collection and questionnaires required for the study; (4) were not currently receiving, or had received, immunological treatment for allergens; (5) did not have severe respiratory diseases, immunodeficiency diseases, cardiovascular diseases, or other chronic diseases; and (6) the child's guardian was fully aware of the purpose of the study and signed written informed consent. When enrolling participants for the study, we conducted a detailed collection of medical history and physical examination for each child to ensure they met the aforementioned inclusion criteria.

The diagnosis of asthma, and atopic dermatitis was further carried out by experienced clinicians in the included children according to the appropriate guidelines^14,15^. A total of 1,405 children with rhinitis were finally included, including 1,030 (73.3%) cases of rhinitis alone, 300 (21.4%) cases of rhinitis combined with asthma, 75 (5.3%) cases of rhinitis combined with dermatitis, and 1 case of combined asthma and dermatitis (due to the small sample size of this group, it was not included in the subsequent analysis).

This study and the use of the participants' biological samples were approved by the Ethics Committee of the First Affiliated Hospital of Guangzhou Medical University (GYYY-2020-73). All participants signed written informed consent independently or by their parents (for children).

## Method

### Collection and processing of blood samples

Extract venous blood from each participant into a yellow-capped tube (5 mL) containing a anticoagulant. After standing at room temperature for 1 h, it was centrifuged at 1000×g for 10 min, and extract the upper serum sample at a rate of 500 μL/tubes are divided into frozen storage tubes (SORFA, Zhejiang, China) and stored in a – 80 °C refrigerator for unified testing of allergen sIgE to avoid repeated freeze–thaw.

### Allergen sIgE detection

Allergen sIgE test kits based on capture enzyme-linked immunosorbent assay (BioCLIA ® 500, Suzhou HOB Biotechnology Co., Ltd. China) were used for the detection of total IgE and serum sIgE of 11 inhalant allergens in all samples. The 11 inhalation allergens included *Dermatophagoides pteronyssinus* (Der p), *Dermatophagoides farina* (Der f), *Ambrosia artemisiifolia* (Amb a), *Artemisia vulgaris* (Art v), *Humulus scandens* (Hum s) *Platanus acerifolia* (Pla a), *Pinus radiata* (Pin r), *Cupressus sempervirens* (Cup s), *Salix capea* (Sal c), *Populus deltoides* (Pop d), *and Betula verrucosa* (Bet v).

In the quantitative detection of serum sIgE antibodies using the capture enzyme-linked immunosorbent assay, antibodies against human IgE are coated on the surface of microplate wells to capture IgE antibodies in the serum. After washing away the remaining serum, the IgE remains bound to the solid phase. Allergen-biotin is then added and incubated in the microplate wells. After another wash, the fixed allergen binds with horseradish peroxidase-streptavidin (HRP-SA) conjugate, forming a sIgE/allergen-biotin/HRP conjugate complex. The wells are washed again, and then incubated with 3,3′,5,5′-Tetramethylbenzidine (TMB) substrate, turning the reaction mixture blue. Finally, sulfuric acid is added to stop the reaction, turning the mixture yellow. The concentration of sIgE in patient samples is proportional to the optical density, which is measured at 450 nm (with a reference wavelength of 620 nm or 630 nm). A standard curve is drawn using the four-parameter method with known concentrations of calibrators, and the concentration of sIgE antibodies is determined using this standard curve.

The levels of specific IgE (sIgE) and total IgE (tIgE) are expressed in kilo units of antibody per liter (kUA/L), with detection ranges of 0.35–100kUA/L and 2–5000 kUA/L, respectively. According to the Radioallergosorbent Test (RAST) grading criteria^16^, an sIgE level less than 0.35 kUA/L is defined as negative, while levels equal to or greater than 0.35 kUA/L are defined as positive.

### Questionnaire

The Standard Questionnaire for Epidemiological Survey of AR in Preschool Children (ESARPC) was designed based on the ISSAC by a team of allergists from the First Affiliated Hospital of Guangzhou Medical University and the National Respiratory Medicine Center, and the quality of the questionnaire was reviewed and evaluated by several experts in the field. The questionnaire included children's demographic information and family history of allergy, as well as symptom scores for the children's nose (nasal congestion, itchiness, runny and sneezing) and eyes (eye itching, hyperemia of conjunctiva, eye swelling and weeping), with each symptom divided into four levels of severity: none, mild, moderate, and severe, corresponding to scores of 0–3. Firstly, the receiving doctor introduced the background of the study, the purpose of the study, and the possible risks and benefits of participating in this program to the patients who met the enrolment criteria and their guardians, and after signing the informed consent for the program, the questionnaires were administered by a doctor or a trained nurse face-to-face with the children, and those who had difficulties in completing the questionnaires were asked to fill them out on their behalf by their guardians. Finally, the questionnaires were collected and verified by the medical staff, and those who did not fill in the questionnaires or omitted to fill in the main content were guided by the medical staff one-to-one on the spot to fill in the questionnaires.

### Definition

#### China's regional division standards

According to climate and geomorphology^17^, it can be divided into Eastern monsoon region, Northwest arid and semi-arid region and Qinghai Tibet Cold region. According to the characteristics of comprehensive geography, natural geography, and human geography^18^, it can be divided into Northern region, Northwest region, Qinghai Tibet region and Southern region. According to the level of economic development^19^, it can be divided into Northeast region, Eastern region, Central region and Western region. According to comprehensive geography^20^, it can be divided into Northeast China, East China, North China, Central China, South China, Southwest China and Northwest China.

### Statistical analysis

The laboratory test results and questionnaire data were organized using Excel 2019 (Microsoft ® Excel ® 2019). Data analysis was performed using GraphPad Prism 8.0.2 (© 1992–2019 GraphPad Software, Inc.), and R Studio 2022.07.2 Build 576 (© 2009–2022 RStudio, PBC). Image modifications and integrations were carried out using Adobe Illustrator CC 2015.0.0 (Adobe Inc.). Binary data were presented as frequency (percentage) and compared across groups using the Chi-square test (X2), with Fisher's exact test employed for calibration when expected frequencies were less than 5. Post-hoc pairwise comparisons were adjusted using the Bonferroni method to correct the significance level. Normally distributed continuous data were represented as mean ± standard deviation, while non-normally distributed data were shown as median (interquartile range), with group differences analyzed using the t-test or Mann–Whitney U test. One-way ANOVA or Kruskal–Wallis H test was used for comparing distributions across multiple groups. Bar graphs were utilized to display percentages. Radar charts were employed to illustrate the prevalence of allergens across various regions. Venn diagrams (Upset R package) were used for visualizing co-sensitization scenarios among different allergens. Violin plots and box plots were applied to display the distribution of nasal and ocular symptom scores among groups. All P-values were based on two-sided tests, and P < 0.05 were considered statistically significant.

### Ethical approval

The study was conducted in accordance with the Declaration of Helsinki, and approved by the Ethics Committee of the First Affiliated Hospital of Guangzhou Medical University (GYYY-2021-67). All participants (children) through their parents, provided their written informed consent.

## Results

### Characteristics of the research objects

A total of 1405 children with rhinitis, aged 5.0 (4.0, 6.0) years, were included in this study, and the overall sex ratio was higher for boys than girls (62% vs. 38%). Patients were mainly from East China (29%), Central China (29%), Southwest China (12%), and South China (11%).The prevalence of sensitization to mite (Der p and/or Der f) allergens in the 1405 children was 39%, and hat to pollen (any one or more types of pollen) was 17%, and the overall tIgE level was 159.6 (39.3, 573.1) kU_A_/L. 1405 children were categorized into three groups based on symptoms: rhinitis, rhinitis combined with asthma, and rhinitis combined with dermatitis, and the differences in demographic information among these three groups were compared (Table 1). The results showed that children in the rhinitis combined with dermatitis group were slightly younger than those in the rhinitis and rhinitis combined with asthma groups (4.0 vs. 5.0 vs. 5.0, *P* < 0.001), and the proportion of boys in the rhinitis combined with dermatitis group was lower than that in the rhinitis and rhinitis combined with asthma groups in the sex ratio (47% vs. 62% vs. 65%). There were no statistically significant differences between the three groups in terms of family history, geographical distribution, season of enrolment and sensitization (*P* > 0.05).

**Table 1:** Demographic characteristics of allergic rhinitis in preschool children.

Variable	N	Overall, N = 1,405	AR, N = 1,030	AR&AS, N = 300	AR&AD, N = 75	p-value
Age	1,405					** < 0.001**
Median (IQR)		5.0 (4.0, 6.0)	5.0 (4.0, 6.0)	5.0 (4.0, 6.0)	4.0 (2.0, 6.0)	
Sex, (%)	1,405					**0.014**
F		539 (38%)	394 (38%)	105 (35%)	40 (53%)	
M		866 (62%)	636 (62%)	195 (65%)	35 (47%)	
Family history, (%)	1,405	971 (69%)	730 (71%)	194 (65%)	47 (63%)	0.057
Region, (%)	1,405					
Northeast		122 (8.7%)	92 (8.9%)	24 (8.0%)	6 (8.0%)	
North		14 (1.0%)	14 (1.4%)	0 (0%)	0 (0%)	
Eastern		411 (29%)	233 (23%)	145 (48%)	33 (44%)	
South		151 (11%)	120 (12%)	29 (9.7%)	2 (2.7%)	
Central		411 (29%)	322 (31%)	75 (25%)	14 (19%)	
Northwest		129 (9.2%)	124 (12%)	4 (1.3%)	1 (1.3%)	
Southwest		167 (12%)	125 (12%)	23 (7.7%)	19 (25%)	
Entry season, (%)	1,405					0.500
Spring		697 (50%)	520 (50%)	142 (47%)	35 (47%)	
Autumn		708 (50%)	510 (50%)	158 (53%)	40 (53%)	
sIgE, (%)						
mite	1,383	545 (39%)	405 (40%)	120 (40%)	20 (27%)	0.069
pollen	1,405	236 (17%)	164 (16%)	61 (20%)	11 (15%)	0.200
tIgE, median (IQR)	1,405	334 (361)	337 (363)	341 (362)	261 (320)	0.300

Proportions were compared between groups using the chi-square test (χ^2^), and for nonparametric quantitative data, the Kruskal–Wallis H test was used for between-group comparisons. If the difference between groups was statistically significant, it was labeled using bold font.

### Distribution of sensitization rates of inhalant allergens in different groups of children with rhinitis

**Figure 1:**
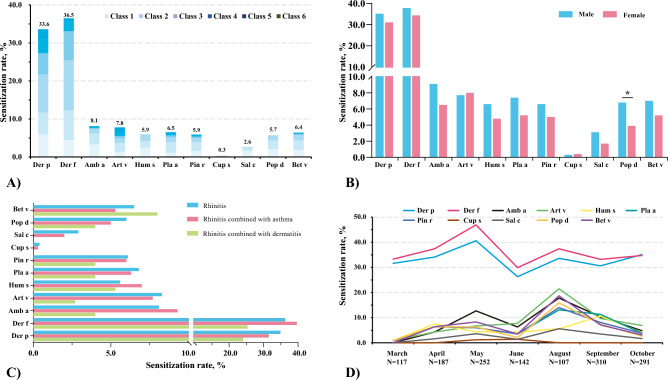
Sensitization rates of mite and pollen allergens in children with rhinitis, showing **(A**) overall sensitization rates, and differences in sensitization rates by (**B**) gender, (**C**) different rhinitis comorbidity groups, and (**D**) different months."

Overall, Der f (36.5%) and Der p (33.6%) were the main allergens of preschool AR children, and Amb a has the highest positive rate among the pollen allergens (8.1%), followed by Art v (7.8%). Allergen sIgE sensitization levels were mainly concentrated in levels 1–3 (Fig. 1A). In terms of gender differences, the sensitization rate of Pop d in boys was higher than that in girls (6.8% vs. 3.9%, *P* = 0.024), whereas there was no significant difference in sensitization rates of other allergens between boys and girls (*P* > 0.05) (Fig. 1B). In addition, we also analyzed the distribution of allergens in children with rhinitis combined with other allergic diseases (Fig. 1C). Among the three groups of children with rhinitis, rhinitis combined with asthma, and rhinitis combined with dermatitis, the sensitization rate of Der f was the highest (36.3%, 39.6%, and 25.3%), followed by Der p (34.9%, 31.5%, and 24.0%). As for pollen allergens, Art v (8.3%), Amb a (9.3%), and Bet v (8.0%) had the highest sensitization rates in the children with rhinitis, rhinitis with asthma, and rhinitis with dermatitis groups, respectively. There was no significant statistical difference in allergen sensitization distribution among the three disease groups.

Finally, we also analyzed the differences in allergen sensitization rates among different months (Fig. 1D). Der p and Der f remained the highest positive rates of inhaled allergens among children with AR, regardless of month. In our overall cohort, sensitization rates of the two types of mites reached their highest in May (Der f: 46.9%, Der p: 40.6%). Art v and Amb a were the main weed pollen in autumn. Art v reaches a sensitization peak (21.5%) in August, while Amb a has a sensitization peak in May (12.7%) in spring and August (17.8%) in autumn, respectively. Hum had a lower overall positive rate, with the peak of sensitization (10.6%) occurring mainly in September in autumn. Surprisingly, in this study, the peak sensitization of various tree pollen in AR patients mainly occurred in August (Bet v: 18.7%, Pop d: 15.9%, Pin r: 14.0%, and Pla a: 13.1%), and we observed that overall the sensitization rate to tree pollen in autumn was higher than that in spring.

### Differences in allergen distribution in children with AR in different regions of China

Based on different delineation criteria, we categorized the multiple centers included in the study into different regions and analyzed the differences in the distribution of mite and pollen sensitization rates in children with AR across different regions (Table 2). Classification of the included regions into Eastern monsoon and Northwestern arid and semi-arid regions based on climatic and geomorphological characteristics showed that the sensitization rates of Der p (36.3% vs. 3.5%, *P* < 0.01) and Der f (39.4% vs. 2.7%, *P* < 0.01) were significantly higher in the Eastern monsoon region than in the Northwestern arid and semi-arid region, whereas among the pollen allergens, Hum s (13.2% vs. 5.2%, *P* < 0.001), Pla a (11.6% vs. 6.0%, *P* = 0.014), Pin r (13.2% vs. 5.2%, *P* < 0.01), Pop d (11.6% vs. 5.1%, *P* = 0.02), and Bet v (10.9% vs. 5.9%, *P* = 0.027) sensitization rates were significantly higher in the Northwest arid and semi-arid region than in the Eastern monsoon region.

**Table 2:** Comparison of sensitization rates of mite and pollen allergens in children with AR between different regions in China.

	Eastern Monsoon	Arid and semi-arid northwest	*P* value	Northeast	East	West	Central	*P* value	Northern	Southern	**Northwestern**	***P* value**
N	1277	129		122	465	394	425		493	784	129	
Der p	461 (36.3)	4 (3.5)	** < 0.001**	40 (32.8)	150 (32.3)	117 (31.4)	158 (37.3)	0.294	121 (24.6)	340 (43.7)	4 (3.5)	** < 0.001**
Der f	501 (39.4)	3 (2.7)	** < 0.001**	51 (41.8)	186 (40.1)	113 (30.3)	154 (36.3)	**0.016**	170 (34.6)	331 (42.5)	3 (2.7)	** < 0.001**
Amb a	102 (8.0)	12 (9.3)	0.611	37 (30.3)	45 (9.7)	15 (3.8)	17 (4.0)	** < 0.001**	87 (17.6)	15 (1.9)	12 (9.3)	** < 0.001**
Art v	101 (7.9)	9 (7.0)	0.863	41 (33.6)	45 (9.7)	11 (2.8)	13 (3.1)	** < 0.001**	94 (19.1)	7 (0.9)	9 (7.0)	** < 0.001**
Hum s	66 (5.2)	17 (13.2)	**0.001**	11 (9.0)	47 (10.1)	18 (4.6)	7 (1.6)	** < 0.001**	58 (11.8)	8 (1.0)	17 (13.2)	** < 0.001**
Pla a	77 (6.0)	15 (11.6)	**0.014**	17 (13.9)	38 (8.2)	21 (5.3)	16 (3.8)	** < 0.001**	59 (12.0)	18 (2.3)	15 (11.6)	** < 0.001**
Pin r	67 (5.2)	17 (13.2)	**0.001**	16 (13.1)	34 (7.3)	23 (5.8)	11 (2.6)	** < 0.001**	53 (10.8)	14 (1.8)	17 (13.2)	** < 0.001**
Cup s	5 (0.4)	0 (0.0)	1.000	1 (0.8)	0 (0.0)	1 (0.3)	3 (0.7)	0.151	1 (0.2)	4 (0.5)	0 (0.0)	0.787
Sal c	32 (2.5)	4 (3.1)	0.566	9 (7.4)	14 (3.0)	4 (1.0)	9 (2.1)	**0.003**	24 (4.9)	8 (1.0)	4 (3.1)	** < 0.001**
Pop d	65 (5.1)	15 (11.6)	**0.002**	16 (13.1)	31 (6.7)	22 (5.6)	11 (2.6)	** < 0.001**	50 (10.1)	15 (1.9)	15 (11.6)	** < 0.001**
Bet v	75 (5.9)	14 (10.9)	**0.027**	28 (23.0)	30 (6.5)	19 (4.8)	12 (2.8)	** < 0.001**	59 (12.0)	16 (2.0)	14 (10.9)	** < 0.001**

Proportions were compared between groups using the chi-square test (χ^2^), and for nonparametric quantitative data, the Kruskal–Wallis H test was used for between-group comparisons. If the difference between groups was statistically significant, it was labeled using bold font. N: Sample size. Der p*: Dermatophagoides pteronyssinus*, Der f*: Dermatophagoides farina*, Amb a*: Ambrosia artemisiifolia*, Art v*: Artemisia vulgaris*, Hum s: *Humulus scandens*, Pla a: *Platanus acerifolia*, Pin r: *Pinus radiata*, Cup s: *Cupressus sempervirens*, Sal c: *Salix capea*, Pop d: *Populus deltoides*, Bet v: *Betula verrucosa.*

In order to assess the differences in the sensitization mites and pollens in children with AR in regions with different levels of economic development, the included regions were divided into the Northeastern, Eastern, Central, and Western regions for comparison. The sensitization rate to Der f was significantly lower in the Western region than in the other three regions (41.8% vs. 40.1% vs. 30.3% vs. 36.3%, *P* = 0.016), and similarly, pollen allergens Amb a (30.3% vs. 9.7% vs. 3.8% vs. 4.0%, *P* < 0.001), Art v (33.6% vs. 9.7% vs. 2.8% vs. 3.1%, *P* < 0.01), Pla a (13.9% vs. 8.2% vs. 5.3% vs. 3.8%, *P* < 0.001), and Cup s (13.1% vs. 7.3% vs. 5.8% vs. 2.6%, *P* < 0.01) were also significantly more sensitizing in the Northeast than in the other three regions, whereas Hum s had a higher sensitization rate in the East and Northeast than in the Central and Western regions. Sensitization rate was higher than in the Central and Western regions (10.1% vs. 9.0% vs. 4.6% vs. 1.6%, *P* < 0.01).

The combined natural and human geographic characteristics were used to comparatively analyze the distribution of mite and pollen sensitization in the Northern, Southern, and Northwest regions. Der p and Der f had higher sensitization rates in the Southern region than in the Northern and Northwest regions (Der p: 43.7% vs. 24.6% vs. 3.5%, Der f: 42.5% vs. 34.6% vs. 2.7%, all *P* < 0.01). Whereas Amb a and Art v had the highest sensitization rates in the Northern region followed by the Northwest region, and the grass pollen sensitization rate was low in the Southern region (Amb a: 17.6% vs. 9.3% vs. 1.9%, Art v: 19.1% vs. 7.0% vs. 0.9%, all *P* < 0.01). The rate of Hum s sensitization in children with AR was significantly higher in the Northwest and Northern than in the Southern (13.2% vs. 11.8% vs. 1.0%, *P* < 0.01). Regarding tree pollen, the sensitization rate to tree pollen was generally higher in the Northern and Northwest regions than in the Southern region.

**Figure 2:**
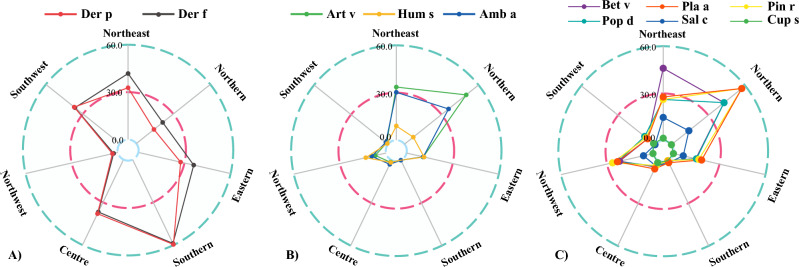
Radar map of the regional distribution of sensitization to (**A**) mites, (**B**) grass pollen and (**C**) tree pollen in children with rhinitis.

The included regions were divided into Northeast, East China, North China, Central China, South China, Southwest, and Northwest based on comprehensive geography. Der p was mainly distributed in South China (59.6%), Central China (38.0%), and Southwest China (37.0%), and Der f were mainly distributed in South China (58.9%), Northeast China (41.8%), Central China (36.8%), and Southwest China (36.4%) (Fig. 2A); Art v and Amb art were mainly distributed in Northeast and North China (Art v: 33.6% and 50.0, Amb a: 30.3% and 35.7%), while Hum s had higher sensitization rates in Northwest and East China (Fig. 2B). Bet v was mainly distributed in Northeast (23.0%) and North China (21.4%). Pla a was mainly distributed in North China (28.60%) and Northeast China (13.9%). Pin r was mainly distributed in North China (28.6%), Northwest China (13.20%) and Northeast China (13.1%) (Fig. 2C).

### Correlation analysis between mites and pollen allergens

**Figure 3:**
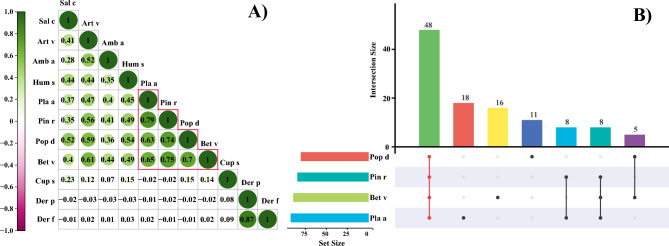
(**A**) Heatmap illustrating the correlation between mites and pollen, and (**B**) an Upset plot reflecting the cross-overlapping of Pla a, Pin r, Pop d, and Bet v.

We analyzed the correlation between mites and pollen (Fig. 3A), and the results showed that mites were not significantly correlated with any of the pollen allergens involved in this study. Der p and Der f had a strong positive correlation (r_spearman_ = 0.87), and the correlation coefficients between pollens (except for Cup s) ranged from 0.28 to 0.79. The correlations among the three grass pollens ranged from 0.35 to 0.52, while among the tree pollens the correlations were stronger between Pla a, Pin r, Pop d and Bet v (r_spearman_ = 0.63 ~ 0.79). Notably, Cup s correlated poorly with all pollen allergens. Co-sensitization of Pla a, Pin r, Pop d, and Bet v was further analyzed (Fig. 3B), and 53.9% (48/89) of Pla a, Pin r, Pop d and Bet v crossed over.

### Comparison of the severity of nasal and ocular symptoms in different groups of AR patients

**Figure 4:**
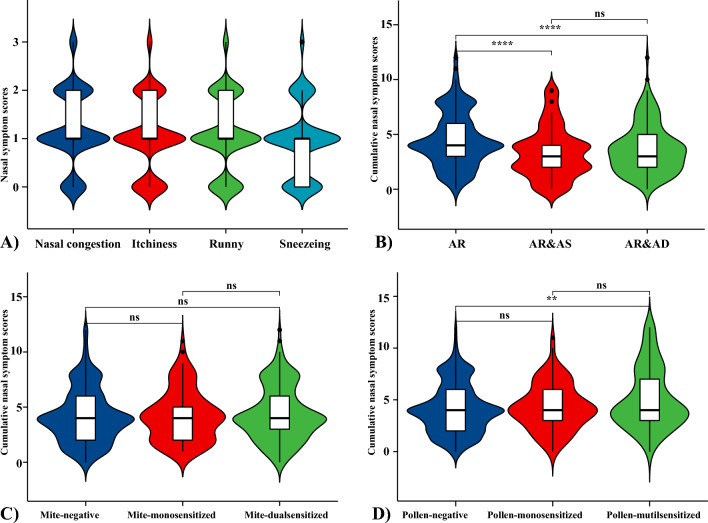
(**A**) Scores for individual nasal symptoms in children with AR, (**B**) the differences in cumulative nasal symptom scores among different rhinitis comorbidity groups, (**C**) the differences in cumulative nasal symptom scores among different mite sensitization groups, and (**D**) the differences in cumulative nasal symptom scores among different pollen sensitization groups. The cumulative nasal symptom score refers to the total score obtained when the severity of nasal congestion, itchiness, runny and sneezing is reported as no symptoms (0 points), mild (1 point), moderate (2 points), or severe (3 points).AR: Allergic rhinitis, AR&AS: Allergic rhinitis combined with asthma, AR&AD: Allergic rhinitis combined with dermatitis. **: *P* < 0.01, *****: *P* < 0.0001, ns: no significance.

The overall nasal symptoms in children with rhinitis were mild, with median scores for nasal congestion, itchiness, runny and sneezing of 1 (1, 2), 1 (1, 2), 1 (1, 2), and 1 (0, 1), respectively (Fig. 4A). The cumulative score of nasal symptoms in children with rhinitis alone was higher than that in the rhinitis combined with asthma group [4 (3, 6) vs. 3 (2, 5)] (*P* < 0.01) and the rhinitis combined with dermatitis group [4 (3, 6) vs. 3 (2, 4)] (*P* < 0.01) (Fig. 4B). On the other hand, the effect of mites or pollen on rhinitis symptoms was further analyzed. There was no statistically significant difference in the cumulative nasal symptom scores among mite-negative, mite-mono-sensitized, and mite-double-sensitized children (Fig. 4C), whereas the cumulative nasal symptom scores were higher in pollen-multi-sensitized children than in pollen-negative children ([4 (3, 7) vs. 4 (2, 6)], *P* < 0.01) (Fig. 4D).

Ocular symptom profiles were also analyzed, and overall ocular symptom scores were low, with an itchy eye score of 1 (0, 1) and median scores of 0 for conjunctival congestion, ocular swelling, and tearing (Supplementary Figures). Children with allergic rhinitis alone had significantly higher cumulative ocular symptom scores than those in the combined asthma and combined dermatitis groups [1 (0, 2) vs. 0 (0, 2)]; and the mite dual sensitization group had higher cumulative ocular symptom scores than the mite-negative group(median (IQR): 1 (0, 2) vs. 1 (0, 2), mean (SD): (1.60 (2.05) vs. 1.47 (2.13), *P* < 0.01), whereas the pollen multi sensitization group had higher ocular symptom scores than the pollen monosensitization and pollen negative groups [1 (0, 4) vs. 1 (0, 3) and 1 (0, 2), *P* < 0.01].

### Analysis of the difference between children with PAR and SAR

**Figure 5:**
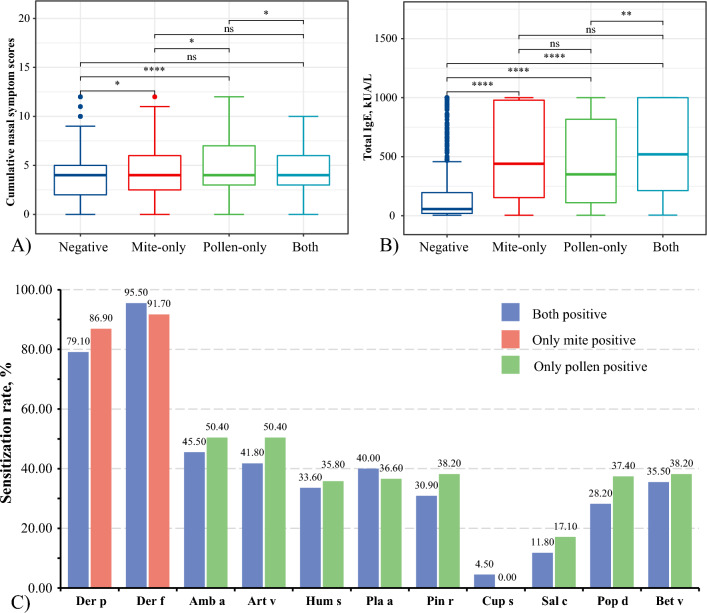
Comparison of mite- and pollen-allergic children with rhinitis for (**A**) differences in nasal symptoms, (**B**) differences in tIgE levels, and (**C**) differences in the positivity rate for all allergens. *: *P* < 0.05, **: *P* < 0.01, **: *P* < 0.0001, ns: no significance.

The cumulative score of rhinitis symptoms in children with only pollen-sensitized was higher than that in the all-negative group [4 (3, 7) vs. 4 (2, 5), *P* < 0.001] and the only mite-sensitized group [4 (3, 7) vs. 4 (2.5, 6), *P* < 0.05]. At the same time, the only mite-sensitized group was also higher than the all-negative group [4 (2.5, 6) vs. 4 (2, 5), *P* < 0.05], while the pollen- and mite-both-sensitized group was lower than the simple pollen sensitization group [4 (3, 6) vs. 4 (3, 7), *P* < 0.05] (Fig. 5A). Further observation of the tIgE levels (Fig. 5B) showed that the tIgE levels in the mite-only, pollen-only, and all sensitized groups were higher than those in all negative groups [440.4 (152.9, 979.1), 350.2 (109.8, 817.1), and 519.9 (212.1, 1000.0) vs. 56.3 (19.5, 195.8, all *P* < 0.01]. There was no statistical difference in the allergen sensitization rates between the mite and pollen-only sensitized, mite-only sensitized, and pollen-only-sensitized groups (Fig. 5C).

For ocular symptom scores, all-sensitized or either mono-sensitized group were higher than in the all-negative group [1(0, 3), 1(0, 4.5) and 1(0, 2) vs. 1(0, 2), all *P* < 0.01]. In addition, the only pollen-sensitized group had higher scores than the only mite-sensitized children [1(0, 4.5) vs. 1(0, 2), *P* < 0.01] (Supplementary Figures).

## Discussion

The prevalence of allergic diseases is increasing in most countries worldwide, and sensitization to mite and pollen allergens is a risk factor for the development of respiratory allergy^21–23^. In this paper, we take children aged 2–7 years old with preschool AR in multi-centers across China as the research participants and investigate and analyze the distribution characteristics of inhalant allergens, regional differences, and population symptom differences in childhood AR, which is a beneficial supplement to the existing allergen atlas of preschool children with AR, and makes up for the shortcomings of the existing studies to a certain extent, and at the same time, this can provide the necessary reference basis for healthcare professionals to better manage the children with preschool AR in their local area. The results of this study indicate that mites (including Der p and Der f) are still the most important inhalant allergens causing AR in preschool children and that sensitization to tree and grass pollens is associated with more severe nasal symptoms in children with AR. In addition, we found that sIgE sensitization to pollen allergens showed distinctive patterns of sensitization between different regions and seasons. The reasons for these pattern differences may be related to geographic factors, climatic factors, socioeconomic level, cultural differences and cross-reactivity.

House dust mite (HDM) is the most important source of sensitization for allergic diseases especially respiratory allergy in southern China^24^. Mites are ubiquitous in human habitats and working environments and are a major source of perennial allergens^25^. In our study, unsurprisingly, mite sensitization rates were more stable in spring and autumn and widely distributed throughout the year, all higher than the various pollen allergens. Sensitization rates for Der p and Der f began to rise in March, with peak sensitization occurring in May in both spring months, followed by a fluctuating rise in autumn. Changes in sensitization rates were correlated with the seasonal distribution of mites. Surveys in many parts of China found that mites could be detected mainly from March, and their numbers began to increase from April to May, reaching a maximum in July to September, with the lowest levels of detectable mites in winter^26–30^.

Climate and geography, socio-economic factors, and differences in human characteristics among regions are important factors contributing to geographic variations in mite sensitization. A 2009 Chinese multicenter study of patients with asthma and/or rhinitis reported significant geographic differences in sensitization rates of HDM^31^.

In our data, both the Northern region and the Southern region bounded by the Qinling Mountains-Huaihe River Line had high mite sensitization rates, with the Northwest being the lowest and dust mite sensitization increasing from North to South. The Southern region is dominated by tropical, subtropical monsoon climate, with high temperatures and rainy, warm, and humid summers. The hot and humid environment is favorable for the growth and reproduction of mites. Therefore, the incidence of mite allergy in the southeast coastal areas is the highest, and the symptoms of allergy patients are also more serious. The cold and dry winter in the Northwest is not conducive to the development and reproduction of mites. Studies have reported that dust mite allergen levels in the South are 50 times higher than those in the Northern region^32^, and the sensitization rate in the south is significantly higher than that in the North^31^, and our findings similarly support this finding. The analysis of geographic regions based on the level of economic development showed that only Der f sensitization rates in the Western region were lower than those in the Northeast, East, and Central regions. Studies have shown that the increase in allergies is associated with urbanized lifestyles, and the "hygiene hypothesis" may partly explain the association between improved hygiene and allergic diseases^33^. Due to geographical and ecological constraints, the western region is still lagging, despite its rising level of economic development.

Pollen is also a clinically important airborne allergen^11^. The sIgE sensitization rate to grass pollen in the preschool children with AR in this study ranged from 5.9% to 8.1%, while the sIgE sensitization rate to tree pollen was lower. This result is similar to the results of the SPT performed on patients with allergic diseases in a hospital in Wuhan City in 2010^34^. In addition, Li J et al. conducted SPT on patients with respiratory allergy symptoms (mainly AR and AS) in 17 cities in China, in which the positive rates of Art v and Amb a were 11.3% and 6.5%, and the positive rates of mixed grass pollen and mixed tree pollen were 3.5% and 2.2%, respectively^31^. The results of their investigations were generally consistent with the present study, and a direct comparison could not be made because some of the allergen types and methodologies were not the same.

The onset of hay fever corresponds to the peak of pollen distribution^35,36^. According to the data of pollen monitoring in Beijing, the monthly distribution of pollen content in Beijing throughout the year showed two peaks. The first peak was dominated by tree pollen of Lignum Vulgare, Populus, and Willow from March to May, which accounted for 53% of the total pollen of the whole year, and the second peak was in August to October, which was dominated by pollen of herbaceous grasses such as Asteraceae, Quinoa, and Amaranthus^37^. In contrast, the results of airborne pollen monitoring in Wuhan were different from those in Beijing, as the peaks of pollen dispersal were in March–April in spring and September–October in autumn, with spring pollen still dominated by tree pollen (Mulberry and Sus r), and autumn pollen dominated by herbaceous pollen (Art a, Hum h, ragweed, etc.)^34^. We found that the peak of sensitization to various tree pollens occurred mainly in August in autumn. According to another survey^36^, the average pollen load in spring was significantly higher than in autumn in all China regions, and our data showed that overall the positive rate of pollen was higher in autumn than in spring, which may be due to the dry climate of our autumn, which is conducive to the spread of pollen.

China has a vast territory, with geographical and climatic environments characterized by North, South, East, and West, and a wide variety of plant species^38^. This is the main reason for the significant geographical differences in pollen allergens in China. In our study, the sensitization of children with AR to four common tree pollens and Hum s were mainly distributed in Northwest arid and semi-arid region. There were significant north–south differences in the distribution of sensitization to all pollen allergens (except Cup s, had a very low overall positivity rate). This is mainly affected by the differences in climate and vegetation types between the north and the south^39^. Further analyzing the pollen prevalence in the seven regions, it was found that the sensitization of tree and grass pollen was mainly distributed in North China and Northeast China. By comparing the allergenic pollen species and dispersal patterns in each geographical region of China, it was found that the main allergenic pollens in Northeast China were summer and autumn pollen and Artemisia, with Artemisia pollen occupying the first place in terms of quantity and allergenicity^38,40^. In North China, spring allergenic pollens were dominated by Elm, Pinus, Populus, and Pinus, while autumn pollens were common in Artemisia, Quinoa, and Humulus^41^. The vegetation types in different regions are different, so conducting airborne allergenic pollen surveys according to the characteristics of vegetation distribution in different regions and summarizing the characteristic allergenic pollens in each region can provide a basis for clinically formulating a reasonable allergen screening program, optimizing the combination of allergen testing, and avoiding the waste of resources.

The results of this study regarding the significant correlation between Der p and Der f sensitization reconfirmed that they have some sequence homology^42^. Except for Cup s, positive correlations were found between grass pollens and tree pollens, with significant co-sensitization of Pop d, Pin r, Bet v, and Pla a, and most studies have shown the presence of cross-reactive proteins between tree pollens^43,44^. Eriksson et al. found that sensitization to birch pollen was often associated with other allergens, and caused mainly nasal symptoms in patients^45^. Sensitization to weed pollen was highly correlated with additional sensitization to grass or certain tree pollens^46^. The presence of cross-reactivity and pollen seasons may require patients to tolerate allergic symptoms for a longer period than if they were sensitized alone, thus negatively affecting their health-related quality of life. Our results found no differences in nasal symptom scores between mite-sensitized alone or dual mite-sensitized. However, children with multiple pollen-sensitized had significantly more severe nasal symptoms than pollen-negative, while those with pollen-sensitized alone had more severe nasal symptoms than those with mite-sensitized alone. This suggests that sensitization to pollen will cause severe allergic symptoms in patients or exacerbate nasal and ocular symptoms in patients with mite-induced AR. Previously, it has been shown that pollen concentration has a greater effect on the symptoms of pollen-allergic patients, with higher pollen concentrations being associated with more severe symptoms of allergic rhinitis^34^.

However, it's also imperative to acknowledge certain limitations in our study. Firstly, the study did not investigate the correlation between skin prick test (SPT) and inhalant-specific IgE levels. SPT are pivotal in diagnosing AR, and their correlation with specific IgE levels would provide a more complete allergenic profile. Furthermore, the influence of medication usage and access to healthcare resources on symptom severity in AR was not assessed. This is particularly relevant in a pediatric population, where treatment adherence and healthcare accessibility can significantly impact AR management. Another limitation of this study is the lack of multivariate analysis in examining the epidemiologic data of children with allergic rhinitis in different regions of China, which prevented us from determining the causal relationship between the relevant factors and the disease as well as evaluating the degree of weighting of the various factors.

In conclusion, this multi-centre study offers a comprehensive analysis of sensitization patterns to dust mites and multiple pollen allergens in preschool children. In most regions of China, HDM remains the main source of sensitization to AR in preschool children, and sensitization to pollen exacerbates nasal and ocular symptoms in children with AR. Sensitization to mites and grass pollen exhibits distinct regional disparities, with allergies to grass pollen prevailing during autumn and pollen sensitization exhibiting a distinct seasonal pattern.

### Supplementary Information

Supplementary Information.

## Data Availability

The datasets used and/or analysed during the current study available from the corresponding author on reasonable request.

## Abbreviations

Amb a: *Ambrosia artemisiifolia*
AR: Allergic rhinitis
Art v: *Artemisia vulgaris*
ARC: Allergic rhinoconjunctivitis
Bet v: *Betula verrucosa*
Cup s: *Cupressus sempervirens*
Der f: *Dermatophagoides farina*
Der p: *Dermatophagoides pteronyssinus*
ESARPC: Epidemiological Survey of Allergic Rhinoconjunctivitis in Preschool Children
HDM: House dust mites
HRP-SA: Horseradish peroxidase-streptavidin
Hum s: *Humulus scandens*
IgE: Immunoglobulin E
ISAAC: International Study of Asthma and Allergies in Childhood
PAR: Perennial allergic rhinitis
Pla a: *Platanus acerifolia*
Pin r: *Pinus radiata*
Pop d: *Populus deltoides*
Sal c: *Salix capea*
SAR: Seasonal allergic rhinitis
sIgE: Specific immunoglobulin E
SPT: Skin prick test
TMB: Tetramethylbenzidine
