# Supplementary material for: Multicenter study of seasonal and regional airborne allergens in Chinese preschoolers with allergic rhinitis
Source: Sci Rep. 2024 Feb 27;14:4754. doi: 10.1038/s41598-024-54574-z (PMC10899184; doi:10.1038/s41598-024-54574-z)
Supplement: Supplementary file 1 — Supplementary Information. [file 41598_2024_54574_MOESM1_ESM.docx]

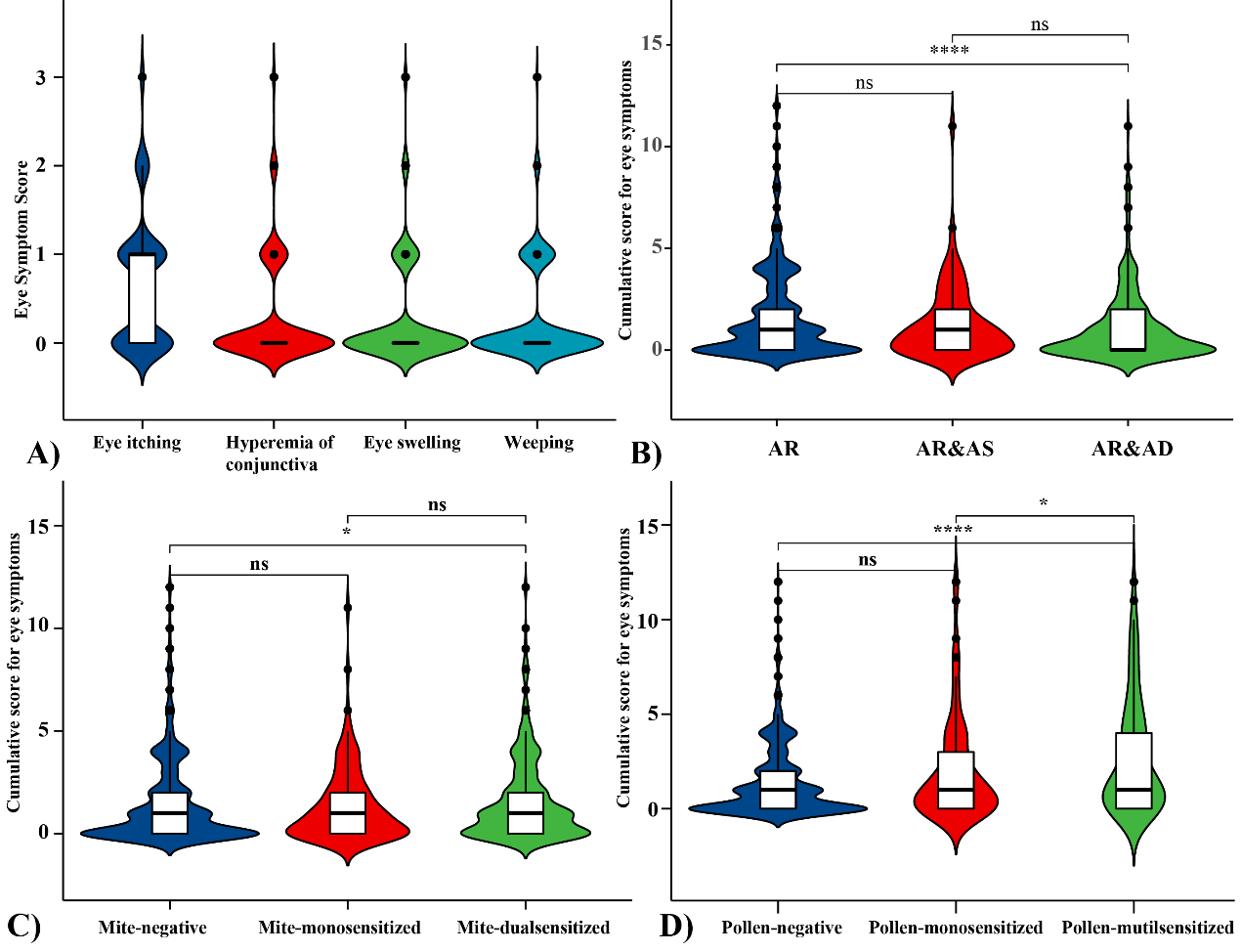


**Figure S1.** (A) Scores for individual ocular symptoms in children with AR (B) the differences in cumulative ocular symptom scores among different rhinitis comorbidity groups, (C) the differences in cumulative ocular symptom scores among different mite sensitization groups, and (D) the differences in cumulative ocular symptom scores among different pollen sensitization groups. The cumulative ocular symptom score refers to the total score obtained when the severity of symptoms like eye itching, hyperemia of conjunctiva, eye swelling and weeping is reported as no symptoms (0 points), mild (1 point), moderate (2 points), or severe (3 points). AR: Allergic rhinitis, AR&AS: Allergic rhinitis combined with asthma, AR&AD: Allergic rhinitis combined with dermatitis. *: *P* <0.05, *****: *P* <0.0001, ns: no significance.


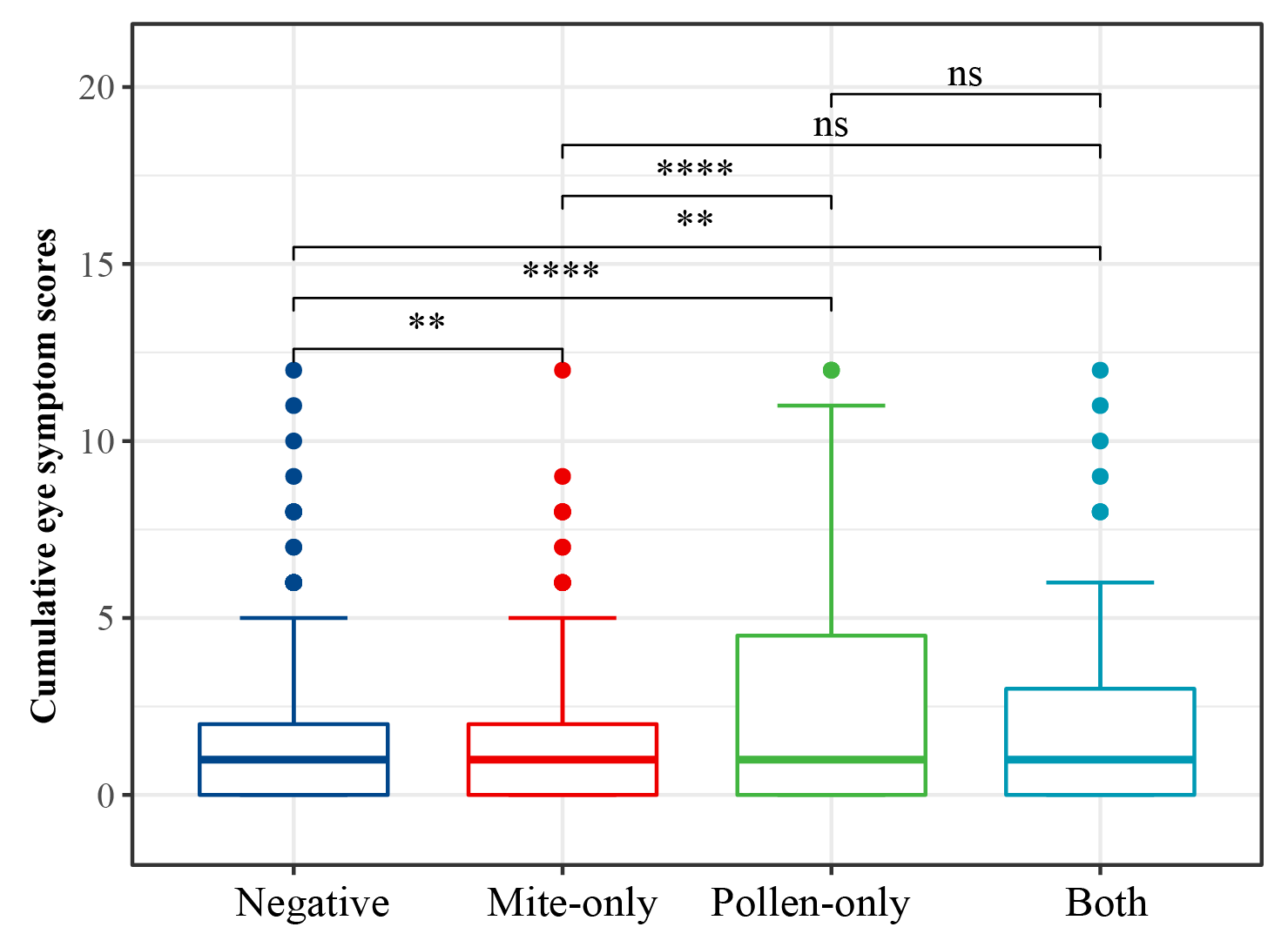


**Figure S2.** Comparison of mite- and pollen-allergic children with rhinitis for differences in ocular symptoms. **: *P* <0.01, *****: *P* <0.0001, ns: no significance.
